# A new experimental approach for studying bacterial genomic island evolution identifies island genes with bacterial host-specific expression patterns

**DOI:** 10.1186/1471-2148-6-2

**Published:** 2006-01-05

**Authors:** James W Wilson, Cheryl A Nickerson

**Affiliations:** 1 Program in Molecular Pathogenesis and Immunity, Department of Microbiology and Immunology, Tulane University Health Sciences Center, 1430 Tulane Avenue, Room 5728, New Orleans, LA 70112 USA

## Abstract

**Background:**

Genomic islands are regions of bacterial genomes that have been acquired by horizontal transfer and often contain blocks of genes that function together for specific processes. Recently, it has become clear that the impact of genomic islands on the evolution of different bacterial species is significant and represents a major force in establishing bacterial genomic variation. However, the study of genomic island evolution has been mostly performed at the sequence level using computer software or hybridization analysis to compare different bacterial genomic sequences. We describe here a novel experimental approach to study the evolution of species-specific bacterial genomic islands that identifies island genes that have evolved in such a way that they are differentially-expressed depending on the bacterial host background into which they are transferred.

**Results:**

We demonstrate this approach by using a "test" genomic island that we have cloned from the *Salmonella typhimurium *genome (island 4305) and transferred to a range of Gram negative bacterial hosts of differing evolutionary relationships to *S. typhimurium*. Systematic analysis of the expression of the island genes in the different hosts compared to proper controls allowed identification of genes with genera-specific expression patterns. The data from the analysis can be arranged in a matrix to give an expression "array" of the island genes in the different bacterial backgrounds. A conserved 19-bp DNA site was found upstream of at least two of the differentially-expressed island genes. To our knowledge, this is the first systematic analysis of horizontally-transferred genomic island gene expression in a broad range of Gram negative hosts. We also present evidence in this study that the IS200 element found in island 4305 in *S. typhimurium *strain LT2 was inserted after the island had already been acquired by the *S. typhimurium *lineage and that this element is likely not involved in the integration or excision of island 4305.

**Conclusion:**

The "clone-and-transfer" approach of evolutionary study identifies genes whose expression patterns indicate the existence of genera-specific regulatory mechanisms that influence the expression of horizontally-transferred DNA sections. The results provide key information that can be used to facilitate the identification of these regulatory mechanisms.

## Background

Genomic islands are sections of bacterial genomes that have been acquired by horizontal transfer and are characterized by one or more of the following criteria: (1) are absent from the identical location in closely related strains or species; (2) contain an altered G/C content as compared to the rest of the genome; (3) inserted adjacent to a tRNA gene "anchoring" site, (4) associated with mobile DNA sequences such as phage, transposon, or plasmid genes or remnants; (5) can be genetically unstable; and (6) contain blocks of genes that work together for a specific function such as molecular transport, specialized metabolism or host cell interactions [1-4]. The functions of many genomic island gene clusters related to specific phenotypes have been studied in great detail for a number of bacteria [2,5,6]. However, the study of the evolution of genomic islands has been mostly performed at the sequence level using computer software to compare bacterial genomic sequences or by genomic DNA microarray hybridization analysis [7-11].

An alternative way to study the evolution of genomic islands would be to clone the island of interest, transfer it to other bacterial hosts, and analyze the expression and function of the genes once the island is established in the new hosts. When studied in this way, we gain information on how a given genomic island has evolved up to the current point in time. This kind of approach would allow the researcher to ask several key questions concerning the evolution of a genomic island that are difficult or not possible to answer with sequence analysis: (1) Have the island genes evolved to be expressed in only the host in which they are found or are they able to be expressed in other bacterial hosts? (2) If the island genes are able to be expressed in other hosts, is this a narrow range of related hosts or a broad range of hosts? (3) Does the island consist of a combination of genes showing different host requirements for expression? (4) Are there regulators that direct expression of island genes in certain hosts that are absent from other hosts? The answers to these questions would give important insight into the evolutionary distribution of regulatory mechanisms that genomic islands "plug-into" upon horizontal transfer to a given bacterial host background. In certain cases, the presence or absence of an expressed and functional genomic island is a determining factor in assigning a particular bacterium to its species, strain type, or evolutionary lineage [4,7,9]. In addition, knowledge gained from answers to the questions above would allow researchers to better manipulate the useful functions contained on genomic islands for beneficial bacterial genetic engineering purposes.

Very few studies have utilized the "clone-and-transfer" approach described above to study genomic island evolution, and these studies have used a narrow range of closely-related strains or species as island hosts [12,13]. In this study, we transfer a *Salmonella enterica *serovar Typhimurium genomic island (island STM4305) that has been cloned onto a broad-host range vector to a wide range of Gram negative gamma- and alpha-proteobacterial hosts. We then test for the expression of 10 of the island genes (contained in different transcriptional units) in the different hosts using a combination of reverse transcriptase-PCR (RT-PCR) and reporter gene promoter fusions. Our results indicate that: (1) island STM4305 contains genes that are able to be expressed in all hosts tested and genes that are not detectably expressed in certain hosts; and (2) the hosts that are unable to detectably express island STM4305 genes belong to more distantly-related alpha-proteobacterial genera. This information provides experimental evidence for the existence of at least two classes of genomic island genes: (1) those that are expressed by mechanisms that have evolved to be utilized in a broad range of hosts; and (2) those that have evolved to be expressed by other mechanisms that are host-specific. The regulatory systems that control the latter category of genes may represent key mechanisms that serve to shape bacterial evolution by affecting the expression characteristics of horizontally-transferred DNA. Genomic island promoter regions that display host-specific activity, such as the ones identified in this study, can be used as tools to identify the regulators that drive their expression and potentially the expression of genes in other chromosomal regions if the regulatory system is global in nature. To our knowledge, this is the first experimental approach to systematically analyze genomic island gene expression in a broad range of gamma- and alpha-proteobacterial hosts. Our present analysis of island STM4305 also allowed us to determine the sequence of the target site of the IS200 element found in this island in *S. typhimurium *strain LT2, and we propose that this element was inserted after the island had already been acquired by the *S. typhimurium *lineage.

## Results

### *S. typhimurium *genomic island 4305

**Figure 1:**
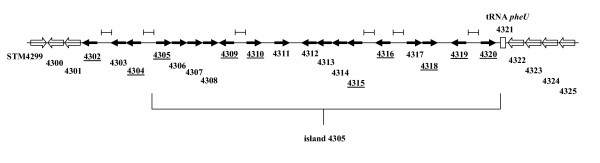
**The *S. typhimurium *genomic island STM4305 region**. A map of the *S. typhimurium *genomic island STM4305 region is depicted. The arrows (both open and black) indicate the genes of the region that have been cloned onto R995 using VEX-Capture. The black arrows indicate genes or operons whose expression has been analyzed using RT-PCR or *lacZ *reporter assays in this study. The genes that comprise island STM4305 are indicated by a bracket below the map. The STM gene number is indicated below each gene. Underlined STM numbers indicate the genes to which primers were designed for RT-PCR analysis. The brackets above the diagram indicate intergenic sections that range in size from 265 to 1658 base pairs and are likely promoter regions. All other regions between the bold arrow genes range in size from -7 to 84 base pairs (except for a 248 bp region between STM4318 and 4319). Note that STM4311, which is present in the sequenced *S. typhimurium *strain LT2, is not present in strains χ3339 and UK1 (see text and Figure 2. The *pheU *tRNA gene (STM4321) is indicated as an open box.

The genomic island inserted at the *pheU *tRNA gene in *S. typhimurium *has been previously identified and is named for the first STM ORF that is present in the island (STM4305) [14,15]. Though the *pheU *tRNA is a common site for insertion of genomic islands in different Gram negative bacteria, the genes of the 15 kb island 4305 appear to be unique to *S. typhimurium *[14]. The genes of island 4305 and adjacent chromosomal genes are listed in Table 2. We previously cloned a 26 kb *S. typhimurium *genomic region that included the entire island 4305 onto the self-transmissible, broad-host-range plasmid R995 using the VEX-Capture technique for targeted cloning of large chromosomal sections [16]. A map of the island and the adjacent genes that are present in the R995 + island 4305 clone is presented in Figure 1. Note that the likely transcriptional units/operons and promoter regions can be predicted from the spacing and orientation of the genes in this region.

**Table 1:** Strains used in this study.

**Strain**	**Relevant characteristics**	**Source and/or reference**
*Salmonella enterica *sevovar Typhimurium		
χ3339	Mouse-passaged SL1344	(17)
χ3339 Δ island 4305	Contains deletion of island 4305	(16)
χ3339 *invA*	*invA*::Km mutation, non-invasive	(This study)
LT2		(15)
UK-1		(17)
*Escherichia coli*		
TOP10		Invitrogen, Carlsbad, CA
TOP10 Rif	Rifampicin resistant	(16)
*Pseudomonas putida*	Prototrophic	ATCC 12633
*Pseudomonas aeruginosa *PAC452	Prototrophic	(35)
*Vibrio vulnificus *M06-24 Rif	Rifampicin resistant	(this study)
*Pseudomonas stutzeri*	Prototrophic	ATCC 17588
*Sphingomonas paucimobilis*	Prototrophic	ATCC 29837
*Novosphingobium capsulatum*	Prototrophic	ATCC 14666
*Agrobacterium tumefaciens *A136	Nalidixic acid resistant	(30)
*Rhizobium meliloti *F34 Nal	Nalidixic acid resistant	(30)
*Rhodobacter sphaeroides *2.4.1	Nalidixic acid resistant	(30)

**Table 2:** Genes of the island STM4305 region

	**STM**		**Common gene**	
	**number**		**name**	**Predicted gene function**
	STM4299		*melB, mel-4*	Sodium, melibiose permease II
	STM4300		*fumB*	Fumarase B (fumarate hydratase class I)
	STM4301		*dcuB, genF*	Dcu family, anaerobic C4-dicarboxylate transporter
	STM4302			Putative cytoplasmic protein
	STM4303		*dcuR, yjdG*	Response regulator in two-component system with DcuS
	STM4304		*dcuS, yjdH*	Sensory histidine kinase in two-component system with DcuR

island genes	STM4305		*dmsA*	Putative anaerobic dimethyl sulfoxide reductase, subunit A
	STM4306		*dmsB*	Putative anaerobic dimethyl sulfoxide reductase, subunit B
	STM4307		*dmsC*	Putative anaerobic dimethyl sulfoxide reductase, subunit C
	STM4308			Putative component of anaerobic dehydrogenases
	STM4309			Putative periplasmic or exported protein
	STM4310			Putative inner membrane protein
	STM4311		*tnpA*	IS200 transposase
	STM4312			Putative phage protein
	STM4313			Putative cytoplasmic protein
	STM4314		*rtsB*	Regulatory protein, LuxR family
	STM4315		*rtsA*	Regulatory protein, AraC family
	STM4316			Putative cytoplasmic protein
	STM4317			Putative helix-turn-helix protein, CopG family
	STM4318			Putative acetyltransferase
	STM4319		*phoN*	Non-specific acid phoshpatase
	STM4320			Putative regulatory protein, MerR family

	STM4321		*pheU tRNA*	*pheU *tRNA
	STM4322		*yjdC, cutA3*	Putative regulatory protein, MerR family
	STM4323		*dsbD, cutA2*	Thiol-disulfide interchange protein
	STM4324		*cutA, cutA1*	Putative periplasmic divalent cation tolerance protein
	STM4325		*dcuA, genA*	Dcu family, anaerobic dicarboxylate transport protein

### The IS200 element present in island STM4305 in strain LT2 is absent from the corresponding location in strains χ3339 and UK-1: a rare glimpse at an IS200 target site before insertion

**Figure 2:**
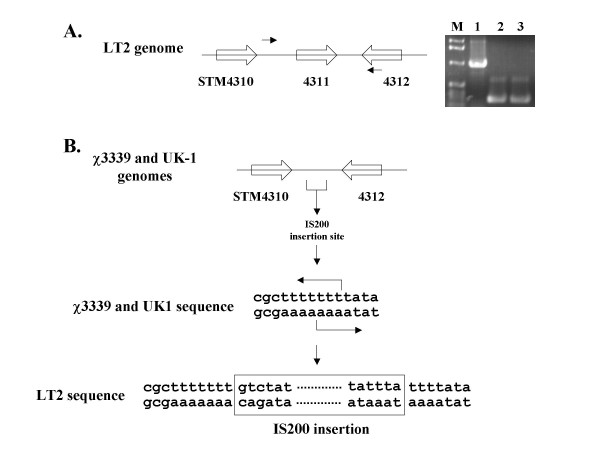
**Characterization of the IS200 region of island 4305**. Panel A depicts the genes STM4310, 4311, and 4312 in *S. typhimurium *island 4305. Gene STM4311 is the IS200 transposase that is present in strain LT2. Small arrows indicate primers that were used to amplify the indicated region between STM4310 and 4312 in strains LT2, χ3339, and UK-1. The DNA agarose gel displays the products from PCR reactions using the indicated primers to amplify chromosomal DNA isolated from the following hosts: lane 1, LT2; lane 2, χ3339; lane 3, UK-1. Lane "M" indicates a DNA size marker. Panel B depicts the STM4310 – STM4312 region in strains χ3339 and UK-1. Sequencing of the χ3339 and UK-1 PCR products allowed the target site of the IS200 element before insertion to be identified. The target site was found to be three "T" residues embedded in an A/T rich region. Note that IS200 is a rare example of an insertion sequence that does not have exact repeats at each end. The sequence of the ends shown here in the LT2 sequence are identical to other previously sequence IS200 elements.

The sequence of island 4305 was obtained from the *S. typhimurium *strain LT2 and indicates the presence of an IS200 element inserted cleanly between the STM4310 and STM4312 genes [15]. The STM4311 gene encodes the transposase for the IS200 element. We cloned island 4305 from a different strain of *S. typhimurium*, χ3339, which is a mouse-passaged isolate of strain SL1344 [17]. PCR analysis of the structure of island 4305 from both LT2 and χ3339 indicated that there was DNA absent from the IS200 region in strain χ3339 (Figure 2A). Primers hybridizing to a region just 3' of STM4310 and to the 3' end of the STM4312 ORF gave an approximately 960 bp product for the LT2 genome as predicted, but a smaller 245 bp product for the χ3339 genome. The smaller PCR product was also observed using these primers with the *S. typhimurium *UK-1 genome (Figure 2A). We sequenced the PCR products obtained from χ3339 and UK-1, and analysis of these sequences revealed that the IS200 element was absent from these strains and that the target site of the IS200 element could be identified. This is significant because the IS200 element is extremely stable and jumps very rarely, and the number of sequenced IS200 target sites before insertion is very rare [18,19]. Our sequence analysis revealed that the target site consisted of three "T" residues in the middle of an A/T rich region, and that this target sequence was duplicated upon insertion (Figure 2B). This is very similar to the small number of IS200 target sites that have been previously characterized, though it has been proposed that IS200 duplicates only 1 – 2 bp upon insertion [18,19]. The ends of the IS200 element shown in Figure 2 that are present in strain LT2 are an exact match to other sequenced IS200 ends [18,19]. Since island 4305 was clearly acquired by the *S. typhimurium *lineage before the different strain variations occurred and since the IS200 element is highly stable once it has inserted, we propose that the IS200 element in island 4305 inserted after the island had already been acquired by *S. typhimurium*. The alternative proposal of loss of the IS200 element after island 4305 acquisition is in our view highly unlikely. This implies that IS200 transposase activity is not involved with integration or excision of island 4305.

### Role of island STM4305 in host cell interactions

**Figure 3:**
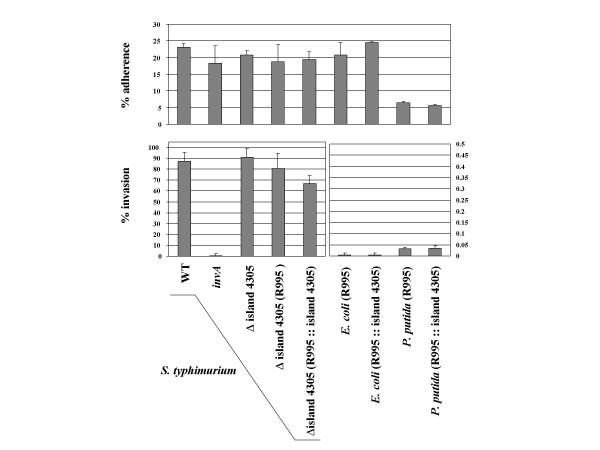
**Adherence and invasion of *S. typhimurium *strains containing a deletion of island 4305 and of *E. coli *and *P. putida *strains containing the cloned island 4305 region**. The indicated bacterial strains were assayed for their adherence and invasion of the HT-29 colonic epithelial cell line as described in the Materials and Methods. The percent adherence is given as the percentage of the inoculum that remained tissue culture cell-associated after washing with buffer. The percent invasion is given as the percentage of adhered bacterial cells that survived gentamycin treatment. The *invA *strain contains a well-characterized mutation that results in severely reduced invasion of *S. typhimurium *into cultured epithelial cells and serves as a control comparison.

The use of the VEX-Capture system to clone island 4305 also allowed convenient *lox*/Cre-mediated construction of an *S. typhimurium *mutant that is deleted for the island 4305 genes [16]. Since a number of *S. typhimurium *genomic islands play a role in the ability of the bacteria to interact with eukaryotic host cells and since the STM4315 gene (*rtsA*) has been previously demonstrated to affect *S. typhimurium *invasion gene expression [20], we compared the Δ island 4305 mutant to the isogenic WT parental strain in the ability to adhere and invade the colonic epithelial cell line HT-29. Our results indicate that deletion of island 4305 has no effect on the ability of *S. typhimurium *to adhere to or invade host cells (Figure 3). We also measured the adherence and invasion of noninvasive *E. coli *and *P. putida *cells containing either R995 or R995 + island 4305 and found that the presence of island 4305 had no effect on the interaction of these bacteria with host cells (Figure 3). We also performed the same assay using J774 murine macrophage-like cells and found that the deletion of island 4305 from *S. typhimurium *and its presence in *E. coli *and *P. putida *did not affect the survival of these strains compared to controls over the course of a 9 hour intracellular survival assay (data not shown). Taken together, these results indicate that island 4305 does not contain any virulence factors that promote interactions with eukaryotic host cells under the conditions tested here.

### Analysis of the chromosomal and cloned island 4305 transcriptional profiles in *S. typhimurium *using RT-PCR

**Figure 4:**
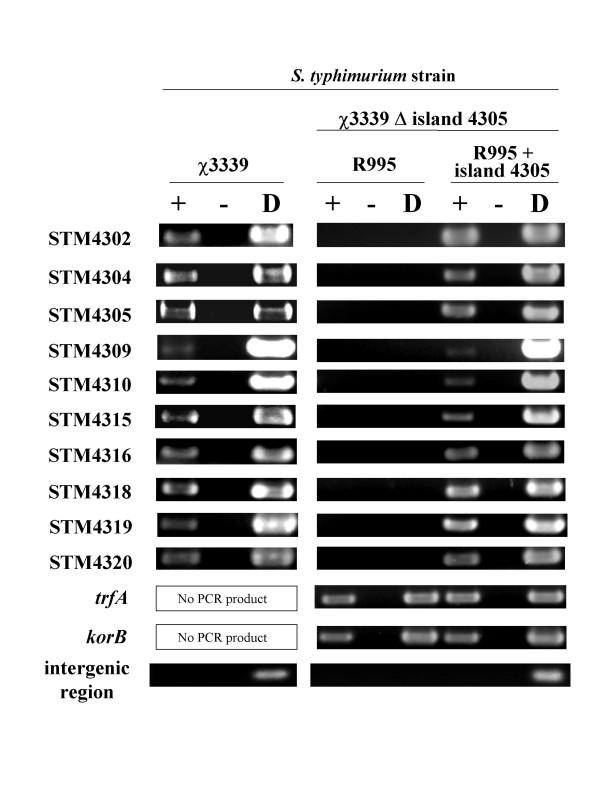
**RT-PCR analysis of chromosomal and cloned island 4305 gene expression in *S. typhimurium***. RT-PCR analysis of island 4305 and adjacent genes in *S. typhimurium *strains χ3339 and χ3339 Δ island 4305 (R995 + island 4305) is depicted. The strain χ3339 Δ island 4305 (R995) was used as a control for the latter strain. The bands shown are from agarose DNA gels that have been stained with ethidium bromide and exposed to UV light. The (+) and (-) lanes represent reactions with and without reverse transcriptase, respectively. The (D) lane represents either χ3339 chromosomal DNA or appropriate plasmid DNA as indicated. The *trfA *and *korB *genes are on the R995 vector plasmid. The intergenic region is a region predicted to be non-transcribed that is located between the STM4304 and 4305 genes.

To analyze the gene expression profile of island 4305 in *S. typhimurium*, we used RT-PCR assays to detect gene transcription. Total cellular RNA was isolated from late-log phase WT strain χ3339 cultures, single-strand cDNA was synthesized, and the cDNA was used as template for PCR amplification using primers specific to the island 4305 genes that are indicated in Figure 1 and that belong to different transcriptional units in the island. We also included two genes just adjacent to island 4305 in our analysis, STM4302 and STM4304. The RT-PCR analysis indicated that all the tested genes contained in island 4305 and the adjacent genes are detectably expressed in *S. typhimurium *under the conditions used here (Figure 4). We then tested the expression profile of the tested genes in the strain χ3339 Δ island 4305 containing the R995 + island 4305 plasmid. The strain χ3339 Δ island 4305 containing R995 was used as a control. We found that the tested genes were detectably expressed from the cloned island in *S. typhimurium *while no RT-PCR products were observed for the tested island genes in the R995-containing strain (Figure 4). However, two genes that are part of the R995 vector, *trfA *and *korB*, were detectably expressed in both the R995 and R995 + island 4305 strains. Primers hybridizing to an intergenic region between genes STM4304 and STM4305 that is predicted to be non-transcribed were also used as a negative control. These primers gave no RT-PCR product in all strains tested, but did give a product when an island-containing DNA template was used (either χ3339 chromosomal DNA or R995 + island 4305 plasmid DNA). The result with gene STM4309 in this analysis is worth particular note. As compared to the other tested genes, STM4309 gave a weakly detectable band, and this result was observed for both the chromosomal and cloned island R995 regions (Figure 4). Overall, the RT-PCR analysis indicates that the transcriptional profiles of the chromosomal and cloned island 4305 regions are extremely similar.

### Transfer of island STM4305 to a range of Gram negative bacteria and analysis of island gene expression by RT-PCR

**Figure 5:**
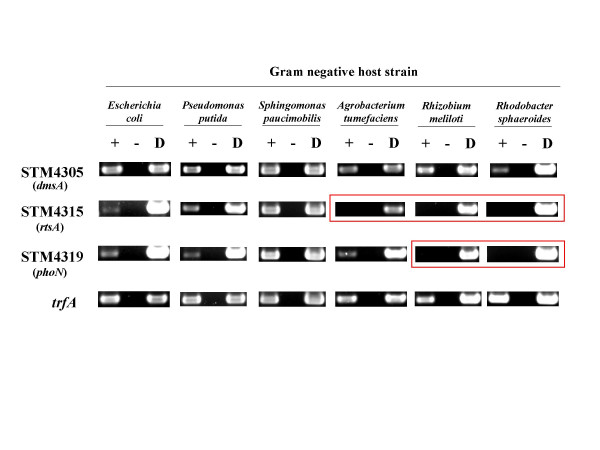
**RT-PCR analysis of cloned island 4305 gene expression in different Gram negative hosts**. Representative examples of RT-PCR analysis of cloned island 4305 and adjacent gene expression in a range of Gram negative hosts is presented. A full catalog of the results is listed in Table 3. Lanes in the agarose gels are as described in Figure 4. The boxed pictures indicate where expression of the tested gene is not detectable. All Gram negative hosts containing the plasmid R995 gave the expected negative results in the RT-PCR analysis (data not shown).

The vector used for the cloned island 4305 region, R995, is a broad-host-range, self-transmissible plasmid. This allowed us to study the *S. typhimurium*-specific island 4305 in a wide range of other Gram negative hosts to gain information on genomic island evolution. We transferred R995 + island 4305 to ten other Gram negative hosts belonging to eight different genera as described in the Materials and Methods. Five of the hosts (*E. coli*, *P. putida*, *P. aeruginosa*, *P. stutzeri*, and *V. vulnificus*) belong to the gamma-proteobacterial group (as does *S. typhimurium*), and five of the hosts (*S. paucimobilis, N. capsulatum, A. tumefaciens, R. meliloti*, and *R. sphaeroides*) belong to the alpha-proteobacterial group. The same strains containing R995 were used for control comparisons. After we obtained the desired transconjugants and confirmed the establishment of R995 + island 4305 in the different hosts, we used the RT-PCR analysis as described above to determine if the tested genes are expressed in the alternative hosts. A representative example of our results is shown in Figure 5 and a full list of the results in each host is shown in Table 3. Some of the tested genes were expressed in all hosts tested (STM4305 in Figure 5 is an example). However, some genes were not detectably expressed in certain hosts while being expressed in others (STM4315 and STM4319 in Figure 5 are examples). The *trfA *gene contained on the R995 vector was strongly expressed in all hosts. Comparing the quantitated RT-PCR signals of the tested genes to that of trfA in each host allowed us to put the genes into three different expression groups. Using a ratio of each gene signal to that of the *trfA *signal, the following groups could be assigned: (1) Genes that were readily detectable had ratios from 1.0 – 0.2; (2) genes that were barely detectable had ratios of 0.2 – 0.02; and (3) genes that were not detectable had ratios of less than 0.02. These results, which can be arranged as a matrix to give an expression "array" of the island genes in the different bacterial backgrounds, are summarized in Table 3. A number of observations can be made by scanning this table: (1) some of the tested genes are expressed in all hosts tested; (2) some of the genes are not detectably expressed in some of the hosts; and (3) the hosts that are not able to express certain genes are not randomly distributed, but are restricted to the more distantly-related genera of the alpha-proteobacterial group. These genera, *Novosphingobium, Agrobacterium, Rhizobium*, and *Rhodobacter*, contain bacteria that are found exclusively in environmental soil and water habitats, as opposed to the other genera that contain bacteria that can be found to interact with mammalian cells.

**Table 3:** Summary of RT-PCR analysis of island 4305 and adjacent gene expression in a range of Gram negative hosts.

		STM gene											
		
		4302	4304	4305	4309	4310	4315	4316	4318	4319	4320	*trfA*	inter*
	*Salmonella typhimurium*	**++**	**++**	**++**	**+**	**++**	**++**	**++**	**++**	**++**	**++**	**++**	**-**
	*S. typhimurium *island 4305 (R995 + island 4305)	**++**	**++**	**++**	**+**	**++**	**++**	**++**	**++**	**++**	**++**	**++**	**-**

**Gamma proteobacterial hosts for (R995 + island 4305)**	*Escherichia coli*	**++**	**++**	**++**	**++**	**++**	**+**	**++**	**++**	**++**	**++**	**++**	**-**
	*Pseudomonas putida*	**++**	**++**	**++**	**++**	**++**	**++**	**++**	**++**	**++**	**+**	**++**	**-**
	*Pseudomonas aeruginosa*	**++**	**++**	**++**	**++**	**+**	**++**	**++**	**++**	**+**	**++**	**++**	**-**
	*Vibrio vulnificus*	**++**	**++**	**++**	**++**	**++**	**+**	**++**	**++**	**++**	**++**	**++**	**-**
	*Pseudomonas stutzeri*	**++**	**++**	**++**	**++**	**++**	**++**	**++**	**++**	**+**	**++**	**++**	**-**

**Alpha proteobacterial hosts for (R995 + island 4305)**	*Sphingomonas paucimobilis*	**++**	**++**	**++**	**++**	**++**	**++**	**++**	**++**	**++**	**++**	**++**	**-**
	*Novosphingobium capsulatum*	**++**	**++**	**++**	**-**	**-**	**+**	**++**	**++**	**+**	**++**	**++**	**-**
	*Agrobacterium tumefaciens*	**++**	**++**	**++**	**++**	**-**	**-**	**-**	**++**	**++**	**++**	**++**	**-**
	*Rhizobium meliloti*	**++**	**++**	**++**	**++**	**-**	**-**	**++**	**++**	**-**	**++**	**++**	**-**
	*Rhodobacter sphaeroides*	**++**	**++**	**++**	**-**	**-**	**-**	**+**	**++**	**-**	**++**	**++**	**-**

"**++**" is an expression ratio of 1.0 – 0.2 compared to *trfA *(easily detectable) as in Materials and Methods; " **+**" is a ratio of 0.2 -0.02 (barely detectable); " **-**"is a ratio of ≤0.02 (i.e. non-detectable)

* Intergenic region predicted to be non-transcribed between STM4304 and 4305.

### Subcloning of the STM4315 and STM4318 promoter regions into a *lacZ *reporter vector

**Figure 6:**
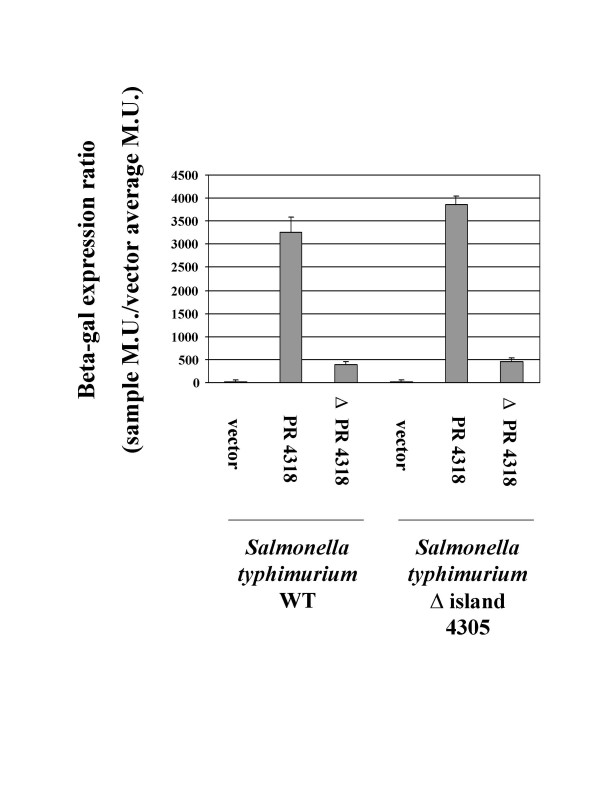
**Expression of PR 4318 and ΔPR 4318 *lacZ *constructs in *S. typhimurium***. The STM4318 promoter region (PR 4318) was subcloned and transcriptionally-fused to a promoterless *lacZ *gene on a broad-host-range plasmid and assayed for beta-galactosidase expression in the *S. typhimurium *hosts χ3339 WT and χ3339 Δ island 4305. The same subcloned fragment but deleted for the predicted STM4318 promoter region (Δ PR 4318) was also used similarly. The results are given as a beta-gal expression ratio as indicated in the Y-axis label and in the Materials and Methods.

**Figure 7:**
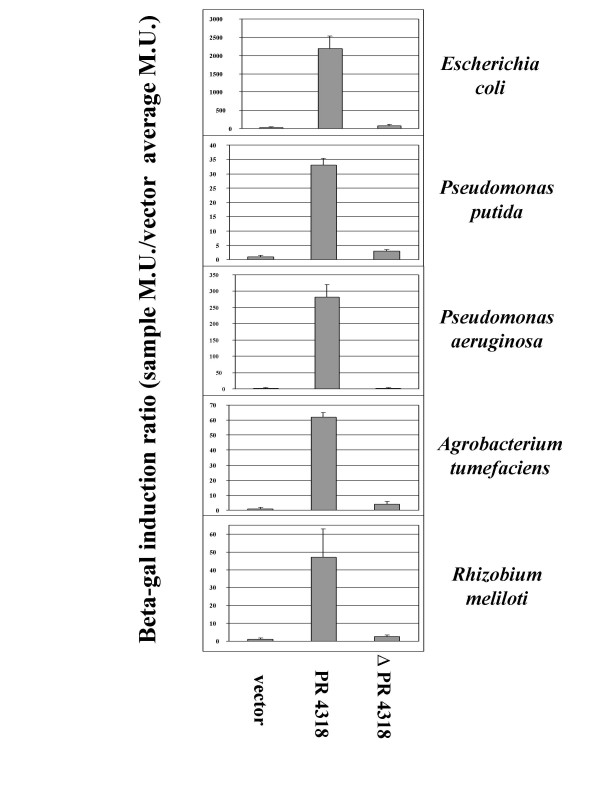
**Expression of PR4318 and ΔPR 4318 *lacZ *constructs in a range of Gram negative host backgrounds**. Analysis of PR4318 and Δ PR4318 lacZ fusion activity was performed as in Figure 6, except that the indicated hosts represent a range of bacterial genera of varying evolutionary relationships to *S. typhimurium*.

To confirm our RT-PCR results and to show the utility of subcloning genomic island promoters to express genes in a host-specific manner, we transcriptionally fused the STM4315 and STM4318 promoter regions (PR 4315 and PR 4318, respectively) to a *lacZ *reporter on an IncQ-based, broad-host-range plasmid. We then transferred these constructs to other Gram negative hosts and assayed *lacZ *expression in each host compared to a vector only control. In the WT and Δ island 4305 *S. typhimurium *hosts, PR 4318 expressed *lacZ *at a very high level indicating that the subcloned promoter region is active and does not require other island 4305 genes to function (Figure 6). We also constructed a *lacZ *fusion in which the STM4318 promoter region was deleted from the subcloned fragment (ΔPR4318) and this fusion displayed significantly decreased *lacZ *activity in both *S. typhimurium *hosts (Figure 6). In the Gram negative hosts *E. coli*, *P. putida*, *P. aeruginosa*, *A. tumefaciens*, and *R. meliloti*, PR 4318 induced *lacZ *expression to levels ranging from 30 – 2000 fold over the vector control (Figure 7). This result supports the RT-PCR findings since STM4318 was found to be detectably expressed in all hosts tested via this assay. The ΔPR 4318 construct displayed significant or complete loss of *lacZ *activity compared to the PR 4318 constructs in all hosts tested (Figure 7).

**Figure 8:**
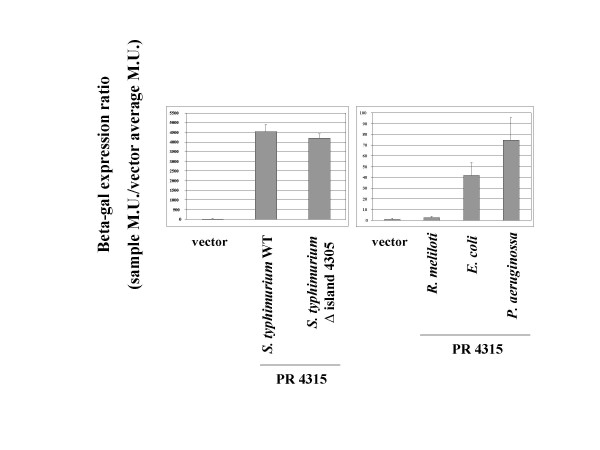
**Expression of PR 4315 *lacZ *construct in *S. typhimurium *and other Gram negative hosts**. The STM4315 promoter region (PR 4315) was subcloned and transcriptionally-fused to a promoterless *lacZ *gene on a broad-host-range plasmid and assayed for beta-galactosidase expression in the indicated *S. typhimurium *strains and other Gram negative hosts. The results are given as a beta-gal expression ratio as indicated in the Y-axis label and in the Materials and Methods.

The activity of the PR 4315 *lacZ *fusion in the WT and Δ island 4305 *S. typhimurium *hosts was also found to be very high compared to the vector control (Figure 8). However, this construct displayed host specific activity in other Gram negative hosts that was consistent with the RT-PCR results (Figure 8). In *E. coli *and *P. aeruginosa*, PR4315 induced significant levels of *lacZ *activity, but in *R. meliloti*, the activity of the PR 4315 construct was very similar to that of the vector control. These findings demonstrate that the subcloned promoter regions can be used to drive host-specific gene expression in a manner that is predicted by the RT-PCR results.

### Identification of a conserved DNA site that is associated with differentially-expressed island 4305 genes

**Figure 9:**
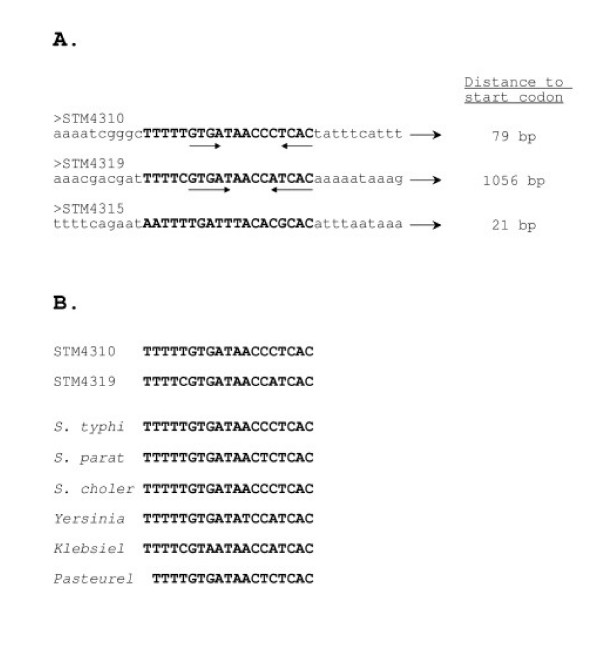
**A conserved DNA site identified in the promoter regions of the STM4310 and STM4319 genes**. Panel A. The DNA sequences of the promoter regions indicated in Figure 1 were aligned and analyzed for common motifs. A 19-bp site was identified in the promoter regions of STM4310 and STM4319, and a related, though divergent, site was found in the STM4315 promoter region. Panel B. The 19-bp site is conserved and found in the genomes of other bacterial species. The sites from the genomes of *Salmonella typhimurium *(STM4310 and STM4319), *S. typhi*, *S. paratyphi*, *S. cholerasuis*, *Yersinia pseudotuberculosis*, *Klebsiella pneumoniae*, and *Pasteurella multocida *are aligned.

To determine if DNA sequence similarities exist between any of the island 4305 promoter regions, these DNA sequences (corresponding to the bracketed regions in Figure 1) were aligned and analyzed for common motifs. Strikingly, a conserved DNA site was identified and found to be located upstream of both STM4310 and 4319 (Figure 9A). A divergent version of this site was also found upstream of STM4315. The STM4310, 4319 and 4315 genes were found to be differentially-expressed. No common DNA site was found to be associated with the genes expressed in all hosts using the methods here. The DNA site is 19 bp in length and differs at only two positions between the STM4310 and 4319 locations. Part of the site displays a palindromic nature as indicated in Figure 9. It is noteworthy to mention that each site is located entirely within the corresponding bracketed regions noted in Figure 1 that contain no open reading frames and are predicted to contain transcriptional signals. A search for this DNA site in genome databases revealed several matches in other gamma-proteobacterial species, including *Salmonella enterica *serovars, *Yersinia pseudotuberculosis*, *Klebsiella pneumonia*, and *Pasteurella multocida *(Figure 9B). We note that these were the only species that gave a significant match to the full 19-bp site and that the sites reside in genomic locations that have not been characterized.

## Discussion

In this report, we present an experimental approach to study the evolution of species-specific bacterial genomic islands that extends the current methods of study. The "clone and transfer" approach presented here involves cloning the genomic island of interest and transferring it to a number of other hosts that represent a range of evolutionary distances from the original host. The researcher can then test island gene expression and function in the alternative hosts. Of particular interest are genes that are either: (1) expressed in the original host but not in alternative hosts, or (2) not expressed in the original host but able to be expressed in alternative hosts. We observed genes belonging to the former class on island 4305 in this study (though STM4309 could potentially belong to the latter class based on its apparent low expression in *S. typhimurium *and apparent higher expression in other hosts). This implies that a mechanism that directs transcription of certain island 4305 genes under the conditions tested here is absent from the hosts in which we are unable to detect expression of these genes. Such regulatory mechanisms are intriguing because their presence or absence in a given host may determine if a particular horizontally-transferred DNA fragment is able to be expressed and to function in that host. Indeed, such regulatory mechanisms could be "molecular keys" that are important factors in shaping bacterial evolution. However, the regulatory mechanisms that control those genes that are expressed in all hosts tested may be significant also. These genes could represent cassettes that are expressed by conserved, highly active sigma 70 (or equivalent analog) promoters or by autoregulatory mechanisms that function in all hosts. These types of mechanisms could yield important information about broad-host-range gene expression systems that allow particular genes to be propagated in a promiscuous manner. Alternatively, the "non-host-specific" genes could be controlled by genetically unlinked regulatory mechanisms that are highly conserved across different bacterial genera.

It is possible that the genes found not to be expressed in certain hosts may be expressed in those hosts under conditions other than those tested here. When testing different growth conditions for use in this study, we found that growth in LB media (at neutral pH) to late log phase was the best condition to allow growth of all the host strains tested so that comparisons across a wide range of bacteria could be made. If there are conditions that allow expression of genes in those hosts where we did not detect expression, clearly the mechanism involved in allowing that expression differs considerably in its regulation and/or activity as compared to in the other hosts where expression was detected under the conditions used here. In addition, an entirely different mechanism could be functioning in those hosts to allow the gene expression under those conditions. In either case, the results would indicate bacterial background-specific differences in the presence or functioning of the regulatory mechanisms that control the gene(s) being analyzed.

Further studies may allow identification of the factors that regulate expression of the host-specific and non-host-specific genes using their promoter regions as an important molecular tool. The identification of such factors could have very important implications. First, certain regulatory mechanisms that control genomic island gene expression are often used by multiple islands (and other non-island genes) in the same host. Therefore, identification of a regulatory mechanism for the genes of one genomic island could represent a global mechanism that is used by that host for multiple different island genes or other genes. There are multiple examples of such regulatory mechanisms, and in many cases, the identification of these mechanisms occurred by identifying differentially regulated genomic island genes [21-25]. Second, these mechanisms could be essential when using genomic islands to engineer alternative bacterial hosts for beneficial medical, environmental, and/or industrial purposes. For example, if a particular host of interest does not express key genes on an island that has been introduced for engineering purposes, then introduction of the necessary regulatory mechanism into this host would then allow the desired gene expression.

**Figure 10:**
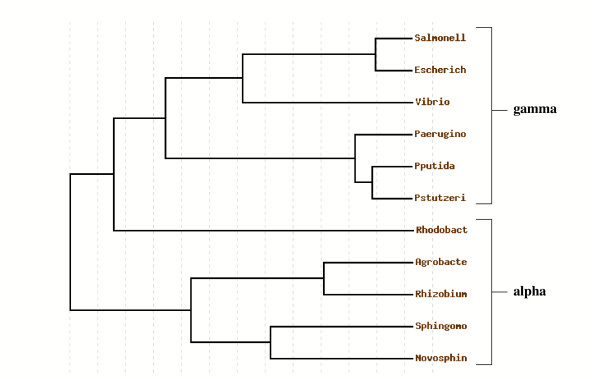
**Phylogenetic tree based on 16S rRNA sequences of the eleven bacterial species used in this study**. The 16S ribosomal RNA sequences of the eleven bacterial species listed in Table 3 were aligned, processed with GBLOCKS to identify conserved segments, and used to construct a rooted phylogenetic tree. The members of the gamma- and alpha-proteobacterial classification groups are indicated.

When analyzing island 4305 gene expression in the other Gram negative hosts, three possible predicted results could be that tested genes are: (1) expressed in all other alternative hosts; (2) differentially expressed in other hosts in a random fashion not dependent on evolutionary relationship; and (3) differentially expressed in other hosts in a manner that displays a pattern based on evolutionary relationship. Our results support the last prediction. This pattern is one such that some island 4305 genes are not expressed in certain hosts, and these hosts belong to more evolutionarily-distant genera of the alpha-proteobacterial group. *S. typhimurium *belongs to the enterobacteriacae in the gamma-proteobacterial group which is characterized by genera that are capable of interacting with mammalian hosts. By contrast, the hosts that were unable to detectably express certain island 4305 genes belong to genera that are characterized by their environmental water and soil habitats and ability to metabolically utilize a wide range of different compounds found in the environment. A phylogenetic tree based on the 16S ribosomal RNA sequences of the bacterial species used in this study confirms that the gamma and alpha classification scheme correlates to basic evolutionary differences in addition to the previously noted phenotypic differences (Figure 10). The tree shows that *S. typhimurium *is indeed more evolutionary related to the other gamma-protobacteria than to the alpha-proteobacteria, as the two classification groups can be clearly divided in the tree. One interesting branch to note is that for *Rhodobacter sphaeroides*; this species has a common ancestor more closely related to the gamma group than the alpha group (though it is still on a branch that is separate from the gamma group). However, this species shows differential expression of island 4305 genes as do other members of the alpha group. The basic differences between the gamma and alpha groups could indicate large differences in the gene regulatory systems that are present in the respective genomes. The identification of these different gene regulatory systems (in this case, those that appear to be present in *S. typhimurium *and absent from *N. capsulatum*, *A. tumefaciens*, *R. meliloti*, and *R. sphaeroides*) may offer clues to the basic genetic regulatory mechanisms that can distinguish different genera over the course of evolution.

Another interesting result from our gene expression analysis is that of the five tested genes that were unable to be detectably expressed in certain hosts, four displayed this result in multiple hosts (i.e. STM4309, 4310, 4315, and 4319). This contributes to the "boxed" pattern of such genes in Table 3. The alternative result of a more random "checkerboard" pattern of such genes in this table was not observed. In our view, this supports the hypothesis that a regulatory mechanism that is conserved in more closely-related hosts is absent from the more distantly-related hosts. Interestingly, STM4315, also known as *rtsA*, is a gene that has been shown (upon overexpression from a plasmid vector) to affect expression of genes that mediate interactions of *S. typhimurium *with host cells [20]. STM4319, known as *phoN*, is a non-specific phosphatase that is found in other members of the enterobacteriacae [26,27]. The genes STM4309, 4310 and 4316 all encode putative, uncharacterized proteins. A conserved 19-bp DNA site was found to be present in the promoter regions of STM4310 and 4319, and a related site was found in the STM4315 promoter region. The site was also found exclusively in the genomes of several other gamma-proteobacterial species. It is not known at this time whether this site plays a role in the differential expression patterns of these island 4305 genes, and further research will be necessary to determine if this DNA sequence plays a role in gene regulation. However, the identification of DNA sites such as this one may provide clues that aid in the characterization of genomic island gene regulation.

The promoters identified in a study such as this one have the potential to be used for experimental applications where the researcher wants a gene turned off (or expressed at very low levels) in one host and turned on upon transfer of this gene to another host (or vice-versa) without the need for the addition of artificial inducers. Such promoters could have significant utility in the study of multi-genera bacterial bio-communities and other broad-host-range bacterial studies. The approach described here allows targeted, systematic identification of such promoters that may be contained on genomic islands or any other region of a bacterial genome.

## Conclusion

The evolution of genomic island genes can be studied by transferring the genomic island to a range of bacterial hosts of different evolutionary distances from the original host and systematically analyzing the expression of the genes compared to proper controls. Using an island cloned from the *S. typhimurium *genome, we show that (1) some island genes are able to be expressed in all hosts tested while some are unable to be detectably expressed in certain hosts; and (2) the hosts that are unable to express island 4305 genes belong to more distantly-related genera of the alpha-proteobacterial group. This provides an approach to answer the basic question of whether genes on the island have evolved to be expressed only in the host of origin or are able to be expressed in other bacterial backgrounds. The information gained from such studies can be used to help facilitate the identification of the mechanisms responsible for any bacteria-specific expression patterns. These potentially important mechanisms may be responsible for determining the expression characteristics of horizontally-tranferred DNA and may serve to globally regulate a number of genes within a given bacterial background.

## Methods

### Bacterial strains and growth conditions

All bacterial strains used in this study are listed in Table 1. Bacterial cells were grown in Lennox broth (LB) [28], LB plus salt [29], YEP [30], YMB [30], M9 minimal media [31], or Burk's minimal media [30] as indicated. Agar was added to a final concentration of 1.5% for all solid media. Antibiotics were used to maintain plasmids in the different host strains as indicated below in the section describing plasmid transfer.

### DNA methods

DNA manipulations were performed using standard protocols as described previously [28,31]. All plasmid DNA was isolated using Qiagen columns as described by the manufacturer (Qiagen, Valencia, CA).

### Cloning of *S. typhimurium *island STM4305

The cloning of the genomic island STM4305 region from the *S. typhimurium *χ3339 genome onto plasmid R995 using the VEX-Capture technique has been described previously [16]. Briefly, *loxP *sites were inserted on both sides of the targeted genomic region, and this region was excised from the chromosome via Cre-mediated site specific recombination as a non-replicating, circular molecule. An R995 plasmid derivative containing a DNA fragment homologous to a small portion of the excised region was used to clone (or "capture") the excised circle via host homologous recombination mechanisms. The R995 + island 4305 plasmid was islolated from *S. typhimurium *via conjugation to a differentially-marked *Escherichia coli *recipient (strain TOP10 Rif). The plasmid-containing *E. coli *strain served as the donor for all the transfers of R995 + island 4305 to other hosts. A spectinomycin/streptomycin marker is present on the cloned genomic region and serves to maintain R995 + island 4305 in the different hosts. The molecular structure of R995 + island 4305 was confirmed in the previously described work [16]. Excision of the targeted chromosomal section created an *S. typhimurium *strain containing a deletion of the island 4305 region (*S. typhimurium *Δ island 4305). This strain was used a host for analysis of R995 + island 4305 in the present studies.

### Transfer of R995 and R995 + island 4305 to other Gram negative hosts

The donor *E. coli *strains TOP10 (R995) and TOP10 Rif (R995 + island 4305) and recipient host strains were grown overnight in separate broth cultures. The cultures were washed with fresh media, mixed at a ratio of approximately 1 donor to 2 recipients, spotted on pre-warmed LB agar plates, and incubated at 30 degrees C for 4 – 16 hours. The mixtures were harvested and transferred to the indicated agar plates containing the indicated selections. The media and antibiotic concentrations used to select for transfer of R995 to each recipient were as follows (concentrations in μg/ml): *P. putida*, M9 kanamycin 50; *P. aeruginosa*, M9 kanamycin 500; *V. vulnificus*, LB salt rifampicin 75, kanamycin 50; *P. stutzeri*, M9 kanamycin 100; *S. paucimobilis*, Burk's tetracycline 5; *N. capsulatum*, Burk's tetracycline 5; *A. tumefaciens*, YEP nalidixic acid 15, kanamycin 50; *R. meliloti*, YMB nalidixic acid 15, tetracycline 15; *R. sphaeroides*, LB nalidixic acid 15, tetracycline 5. The antibiotic concentrations used to select for transfer of R995 + island 4305 to each recipient were as follows (same media as above): *P. putida*, streptomycin 100; *P. aeruginosa*, streptomycin 100; *V. vulnificus*, rifampicin 75, spectinomycin 125 (a rifampicin-sensitive donor was used for this mating); *P. stutzeri*, streptomycin 100; *S. paucimobilis*, spectinomycin 125; *N. capsulatum*, spectinomycin 125; *A. tumefaciens*, nalidixic acid 15, spectinomycin 125; *R. meliloti*, nalidixic acid 15, spectinomycin 125; *R. sphaeroides*, nalidixic acid 15, spectinomycin 125. Plasmid DNA was isolated from each recipient and the presence of island 4305 was confirmed via agarose gel and PCR analysis.

### Tissue culture cell adherence and invasion assays

Adherence to and invasion of the human colonic epithelial cell line HT-29 and survival in the macrophage-like cell line J774.1 were assayed as described previously using 1.5 hour time periods for both the pre- and post-gentamycin incubations [32]. To account for differences in chemotaxis and motility between different genera, all bacterial strains were spun onto the tissue culture cells in a Sorvall table top centrifuge at 1,000 RPM for 5 minutes. There was no difference in motility between the *S. typhimurium *WT and Δ island 4305 strains (data not shown). The data shown are averaged from two to four separate experiments each done in triplicate wells, and the error bars indicate the standard deviation. The invasion data are shown as the percentage of adhered cells that survived gentamycin treatment.

### RT-PCR analysis

PCR and RT-PCR analysis was performed as described previously [16,33,34]. Each RT-PCR experiment was replicated with RNA harvested from two to five separate cultures each grown to late log phase in LB medium. These conditions were found to be best for allowing growth of all the bacterial hosts used in this study so that comparisons of gene expression across a wide range of genera could be made. Each RNA preparation was assessed via gel electrophoresis, and the quality of these preparations was found to be highly reproducible between batches. Reactions were prepared with equivalent amounts of RNA and cDNA, and equal amounts of the reactions were loaded in the depicted agarose gels. RT-PCR DNA bands were quantitated using AlphaEase FC (Alpha Innotech, San Leandro, CA). The RT-PCR expression ratios were calculated by dividing the signal for the test gene band by that of the signal for the *trfA *R995 plasmid vector band for that host. For χ3339, this ratio was calculated similarly but the signal for the STM4318 band was used in place of *trfA *since the *trfA *signal is not present in this host and STM4318 gave an expression ratio of 1.0 (compared to *trfA*) in all hosts tested. All Gram negative hosts containing plasmid R995 gave the expected negative results in the RT-PCR analysis (Figure 4 and data not shown).

### Construction of the STM4315 and STM4318 promoter region *lacZ *fusions

A series of broad-host-range vectors carrying a promoterless *lacZYA *operon was constructed to test promoter activity in a wide range of Gram negative hosts. Details of the construction of these vectors will be provided elsewhere (Wilson JW, Nickerson CA: unpublished results). Briefly, the *lacZ *vector from this series used here, termed pQLacZ1, is derived from the IncQ plasmid vector pJAK13 (ATCC #77288), which is a spectinomycin/streptomycin resistant derivative of pMMB67HE [35]. A *Bam*HI – *Sal*I fragment from pRS415 containing the promoterless *lacZYA *operon [36] was inserted between the *Bam*HI and *Sal*I sites of pJAK13 (such that promoter activity from this vector did not turn on *lacZ *expression) to yield pQLacZ1. A 1.2 kb fragment extending from the 5 ' end of the STM4315 ORF to the 5 ' end of the STM4318 ORF was PCR amplified from the *S. typhimurium *chromosome. *Bam*HI and *Kpn*I sites were added to the primers at the STM4315 and STM4318 ends, respectively. This fragment was cloned into the T/A cloning vector pCR-TOPO4 and verified by sequencing. The fragment was cloned into the *Kpn*I and *Eco*RI sites of pQLacZ1 using the *Kpn*I site of the PCR product and an *Eco*RI site in the pCR-TOPO4 vector such that the STM4318 promoter region (PR 4318) drives *lacZ *expression. The ΔPR 4318 derivative was constructed using an *Eco*RV site upstream of PR 4318 and an *Eco*RI site in the pCR-TOPO4 vector to insert a truncated PCR fragment without PR 4318 into the *Sma*I and *Eco*RI sites of pQLacZ1. The STM4315 promoter region (PR 4315) was inserted into the *Sma*I and *Bam*HI sites of pQLacZ1 using the *Bam*HI and *Eco*RV sites of the PCR fragment such that PR 4315 drives *lacZ *expression. All pQLacZ1 derivatives were mobilized to the indicated hosts using the IncP transfer system of an *oriT*-deficient derivative of pUZ8, termed pUZ8002 (Wilson JW, Figurski DH: unpublished results).

### Beta-galactosidase assays

Beta-galactosidase (beta-gal) assays were performed as described previously using LB as the growth medium for all strains [37]. The beta-gal expression ratio was calculated by dividing the Miller Unit value for each particular sample by the average Miller Unit value obtained for the vector alone in each strain. Thus, this calculation gives the fold induction of beta-gal expression for each strain. This value did not differ significantly from 1.0 for all the vector-containing strains. The data presented are the average from two to four separate experiments each done with triplicate samples.

### Computer analysis methods

To construct the phylogenetic tree, 16S ribosomal RNA sequences of the indicated bacterial species were aligned with the AliBee multiple sequence alignment (a part of the GeneBee internet package), processed with GBLOCKS to identify more highly conserved regions, and organized with the FastDNAml progam [38-41]. Identical results were obtained when the same tree was constructed with the TreeTop program [38]. For promoter analysis, the indicated sequences were aligned and analyzed for common motifs using ClustalW and AliBee and searched against genomic databases using BLAST version 2.2.12 [38,42,43].

## Authors' contributions

JW conceived the project, performed the experimental procedures, interpreted the data and drafted the manuscript. CN participated in the project design, contributed key insight to data interpretation and helped to draft the manuscript. Both authors approved the final draft of the manuscript.
